# Enhancing Performance and Sustainability of Community Health Worker Programs in Uganda: Lessons and Experiences From Stakeholders

**DOI:** 10.9745/GHSP-D-21-00260

**Published:** 2021-12-31

**Authors:** David Musoke, Edwinah Atusingwize, Rawlance Ndejjo, Charles Ssemugabo, Penelope Siebert, Linda Gibson

**Affiliations:** a Department of Disease Control and Environmental Health, School of Public Health, College of Health Sciences, Makerere University, Kampala, Uganda.; b School of Social Sciences, Nottingham Trent University, Nottingham, United Kingdom.

## Abstract

**Background:**

Community health worker (CHW) programs in Uganda have contributed to improved health outcomes in recent years. However, opportunities for engaging the various stakeholders supporting CHW programs have been limited. This article presents workshop findings where several stakeholders shared their lessons and experiences that can enhance performance and sustainability of CHW programs in Uganda.

**Methods:**

We collected qualitative data from stakeholders from government, private, and community organizations, as well as CHWs, involved in CHW programs in Uganda during a 1-day workshop. The workshop involved plenary presentations and group discussions on critical aspects of CHW programs. All proceedings from the workshop were audio-recorded, transcribed, and analyzed by thematic content analysis.

**Results:**

Four major themes emerged from the workshop: lessons learned in implementing CHW programs, challenges affecting CHW programs, performance of CHWs, and ensuring sustainability of CHW programs. Key lessons learned related to 3 main subthemes: capacity building and use of technology, supervision and motivation, and stakeholder engagement and collaboration. Challenges affecting CHW programs identified included poor coordination, fragmented data collection systems, high program expectations, inadequate support mechanisms, and high dropout rates. Mechanisms for improving the performance of CHWs emphasized the need to: strengthen recruitment, training, and retention strategies; improve motivation; streamline coordination mechanisms; and develop and strengthen community health policies. The sustainability of CHW programs requires institutionalization; sustainable funding; economic empowerment of CHWs; local ownership; and a strengthened research agenda.

**Conclusion:**

To improve the performance and sustainability of CHWs programs, stakeholders such as policy makers and implementing partners need to consider CHW needs, existing structures and policies, as well as local support.

## Abstract (precis)

We conducted a 1-day workshop—a unique opportunity to engage stakeholders at all levels of community health worker (CHW) program involvement—to discuss learned experiences and strategies to enhance and sustain the CHW program in Uganda.

## Teaser

**Key Findings:**

- Capacity building and use of technology, supervision, and motivation, as well as stakeholder engagement and collaboration, are key to the success of community health worker (CHW) programs.
- Strengthening recruitment, training, and retention strategies; improving motivation; streamlining coordination mechanisms; and developing and strengthening community health policies are needed to enhance the performance of CHWs.

**Key Implications:**

- Policy makers including the Ministry of Health and implementing partners need to consider CHW needs, existing structures, and policies for enhanced performance of CHWs.
- The workshop methodology can be used in health systems research to inform policy, practice, and programming particularly in low- and middle-income countries, such as Uganda.

## BACKGROUND

Over the past 2 decades, the national community health worker (CHW) program in Uganda,^1^^–^^6^ known locally as village health team members, has been successful especially in the areas of integrated community case management (iCCM) of childhood illnesses, maternal and child health, and HIV/AIDS.^7^^–^^10^ Despite these successes, there have been many missed opportunities for realizing the full potential of CHW programs in Uganda, including lack of consistency in the selection process of CHWs, which creates distrust within communities.^11^ Other health system challenges that affect CHW programs in Uganda include inadequate refresher training, high workload, lack of supervisory support and feedback mechanisms, insufficient remuneration, distrust of CHWs among health care providers, and stock-outs of medicines and supplies.^12^^,^^13^ In addition, funding of the national CHW program has been insufficient, with much of the responsibility left to implementing partners, particularly nongovernmental organizations (NGOs), working across the country. Given these substantial drawbacks, there is a need to strengthen performance and ensure sustainability of CHW programs in Uganda to achieve universal health coverage (Box).

BOX **Community Health Worker Program in Uganda**

In a bid to advance the Alma Ata declaration goal of Health for All,^1^ now popularly referred to as universal health coverage,^2^ the Ugandan health system underwent several reforms.^3^ Among these reforms was the introduction of the community health workers (CHWs) program, locally known as village health teams members.^4^ The criteria the program uses to select community members to be CHWs includes aged 18 years or older, able to read and write in their local language, and has a high level of integrity.^5^ After their initiation training, CHWs engage with communities to identify local health problems and needs, mobilize them for health interventions, and refer and link them to health providers including follow-up. CHWs also collect and maintain records, conduct home visits, treat children aged under 5 years, and provide basic health education.^6^ CHWs are the first contact of the community with the health system and are particularly beneficial in rural areas across Uganda that have limited access to health care. They primarily report to health facilities in their areas that are mandated to provide them with supervisory support.

Several strategies for improving the performance of CHW programs exist globally. An integrated approach where community members work with health care providers and other stakeholders from the public and private sectors in designing programs has been shown to promote joint ownership and improve CHW performance.^14^ Through this approach, stakeholders provide collaborative supervision and constructive feedback, a balanced package of incentives, and practical monitoring using community and health system data.^14^^,^^15^ In fact, the integrated community and health system approach has been reported to reduce workload and increase CHW credibility.^16^ However, fragmentation exists among the national CHW program in Uganda which is partly responsible for the challenges currently being faced. In addition, remuneration of CHWs using a mixture of financial and nonfinancial incentives has been shown to enhance CHW performance and sustainability of CHW programs compared to performance-based financing mechanisms.^16^ Whereas performance-based financing has not been embraced in Uganda, CHWs in the country are volunteers, hence they do not receive any regular remuneration for their services. However, they may occasionally receive some financial and nonfinancial support (such as t-shirts) from partners working with them. Other effective approaches that have been reported to strengthen CHW programs include frequent supervision and continuous training,^16^ which remain inadequate in Uganda.

In Uganda, several stakeholders, including the Ministry of Health (MOH), NGOs, and universities, are involved in and support CHW programs. These stakeholders engage CHWs through recruitment, training, supervision, data collection, motivation, as well as implementation of health programs such as treatment of childhood illnesses, sexual and reproductive health, and control of communicable diseases. However, opportunities for learning across the various stakeholders supporting CHW programs have been minimal, limiting the sharing of lessons and experiences. As such, it is important to engage various stakeholders involved in CHW programming to harmonize approaches that can enhance performance and sustainability. Findings from such engagements can be used by the MOH and implementing partners in Uganda as well as other low- and middle-income countries (LMICs) as they design interventions to improve CHW programs. Although vast evidence exists on CHWs in Uganda,^7^^,^^8^^,^^12^ there is minimal literature on stakeholder experiences in supporting their programs. In addition, the use of workshops to engage participants regarding CHW programs in Uganda (and elsewhere) has hardly been explored. Therefore, we conducted a workshop to explore stakeholders' experiences of implementing and supporting CHWs programs in Uganda to learn of ways in which performance and sustainability of CHWs programs could be enhanced.

Opportunities for learning across the various stakeholders supporting CHW programs have been minimal, limiting the sharing of lessons and experiences.

## METHODS

### Study Setting and Participants

In 2018, we conducted a 1-day workshop in Kampala, Uganda, as part of a project aimed at strengthening the CHW program in Wakiso district, Uganda.^17^ The project was implemented under the partnership between Nottingham Trent University (NTU), United Kingdom, and Makerere University School of Public Health (MakSPH), Uganda.^18^ This workshop was planned independently of other project activities. The workshop was attended by 51 participants who were chosen from government agencies, institutions, and organizations based on their involvement in supporting CHW programs in Uganda, including implementers/NGO staff, researchers/academics involved in CHW programs, students, policy makers from national and subnational levels (Table 1), as well as CHWs from Wakiso district.

**TABLE 1.** Categories of Workshop Participants^a^ on the Uganda CHW Program, N=51

Category	No. (%)
Implementers / NGO staff	19 (37.3)
Researchers / academia	16 (31.4)
Students	7 (13.7)
Policy makers	6 (11.8)
CHWs	3 (5.9)

Abbreviations: CHWs, community health workers; NGO, nongovernmental organization.

a The participants were from Makerere University School of Public Health, Nottingham Trent University, MOH, World Vision, Wakiso District Local Government, Amref Health Africa, Save the Children, Living Goods, Nkumba University, Kabale University, Mbarara University of Science and Technology, FHI 360, Management Sciences for Health, United States Agency for International Development Regional Health Integration to Enhance Services in East Central Uganda Activity, Wise Choices for Life, BRAC, Clinton Health Access Initiative, Africa Community Centre for Social Sustainability, Action for Community Development, Wellshare International, Kampala Capital City Authority, and Mildmay.

### Data Collection

The workshop comprised 3 sessions: (1) presentation of key lessons learned about CHW programs from 4 selected implementing partners, (2) facilitated group discussions on critical aspects of CHW programs; and (3) plenary, where all groups provided feedback from their respective discussion. Each group had an average of 15 people and included policy makers, local government officials, implementers/NGOs, academia, and the community. Details on the specifics of the workshop session are included in the Supplement.

Before the group discussions, the researchers introduced the themes to the workshop participants:

1. Experiences of working with communities for health improvement including what has worked, challenges faced, and how they have been addressed.
2. Enhancing support to CHWs to improve their performance in health service delivery considering their recruitment, training, retention, supervision, motivation, reporting, transportation, equipment and supplies, and use of technology.
3. Increasing sustainability of CHW programs in Uganda considering funding, enabling environment, local government engagement and support, collaboration, monitoring and evaluation, research, innovations, and learning fora.

The researchers distributed themselves among the 3 groups and participated minimally in the discussions to allow other participants to share their experiences. In addition, the researchers did not act as moderators or notetakers for the groups. The researchers clearly described the workshop's purpose, emphasizing that the activity was solely for research, which provided an environment that ensured all participants (especially those from the community) felt comfortable and facilitated sharing of experiences and opinions openly. In addition, researchers stressed that contribution from all participants was important to get the perspectives of the various stakeholders who attended. During the discussions, the group moderators ensured that all members had an opportunity to make contributions, which enabled various participants' perspectives, including those from the community, to be heard. Indeed, the moderators did not allow any group members to dominate the discussions at the expense of others. The moderators intermittently requested contributions from certain members whose views were particularly needed or those who had made minimal input to the discussion. Therefore, the discussions had significant contributions from various group participants including CHWs.

### Data Analysis

The presentations, facilitated group discussions, and other deliberations at the workshop were audio-recorded and transcribed verbatim in English. The notetakers were involved in providing feedback from the groups during the plenary session. The transcripts from the workshop were proofread by 2 experienced researchers in qualitative research and later imported into Atlas.ti. 8.0 for data analysis. Initially, coding was done by the 2 researchers who independently read the transcripts several times and developed codes. The 2 researchers discussed the codes and arising issues and agreed on a coding framework. Thematic content analysis following the semantic approach was used,^19^ and the emerging codes documented. Afterward, all codes were synthesized into emerging subthemes, and subthemes into themes, which are presented in the results supported by quotations.

### Ethical Considerations

This study was approved by Makerere University School of Public Health Higher Degrees, Research and Ethics Committee as part of the project aimed at strengthening the CHWs program in Wakiso district. The study was also approved and registered by the Uganda National Council for Science and Technology. Workshop participants were informed and approved of the use of findings for various dissemination including reports, publications, and conference presentations. All data collected from the workshop were handled confidentially, and participants were kept anonymous.

## RESULTS

Workshop presentations and group discussions highlighted various critical issues in the design, implementation, and evaluation of CHW programs in Uganda. From the analysis of the workshop proceedings, 4 major themes emerged: lessons learned in implementing CHW programs, challenges affecting CHW programs, enhancing performance of CHWs, and ensuring sustainability of CHW programs.

### Lessons Learned in Implementing CHW Programs

Key lessons learned related to 3 main subthemes: capacity building and use of technology, supervision and motivation, and stakeholder engagement and collaboration.

#### Capacity Building and Use of Technology

Stakeholders stressed that capacity building involving regular training and onsite mentorship of CHWs (within communities) is necessary to support their work. They noted that onsite mentorship encouraged the participation of CHWs who did not attend trainings held away from their villages due to transportation challenges. It also emerged that capacity building using technology (e.g., mobile devices) enabled CHWs to perform their roles better and offered benefits, including improved data quality (collection and reporting), helped learn new skills, and motivated and empowered CHWs. With technology and its benefits, stakeholders noted that community health work will continuously advance and can be enhanced if incorporated in the design of CHW programs. However, stakeholders were concerned about the cost implication as well as limited or no mobile phone network coverage in some rural communities, which challenged technology-based interventions and systems.

*…There are many other opportunities which technologies can offer. It comes with a little bit of cost but is worth it. The challenge with technology is some of the rural communities have poor network access which is a huge problem. You have to look for a point where there is a signal before you can do anything with your phone. That is hard in many villages and limits [CHWs'] interest in use of technology.* —Member from an NGO, Group 3

#### Supervision and Motivation

Stakeholders noted that in addition to deployment and training, effective supervision of CHWs remains key. For example, they noted that supervision by CHW parish coordinators was fundamental in linking CHWs to health facilities and enabling delivery of drugs and other supplies. In addition, they reported that regular feedback and collection of reports from CHWs was a key motivation for their performance. They also noted that to motivate CHWs to perform and show appreciation, CHWs were usually given nonfinancial incentives (e.g., certificates and branded t-shirts), which CHWs appreciated. In addition, the availability of adequate equipment and supplies as key inputs for CHWs to be able to continuously respond to community needs and motivation to perform better was strongly emphasized. Stakeholders strongly agreed that while the national CHW program in Uganda was voluntary, financial benefits are also important for enhanced performance to motivate and show appreciation for their services.

*We [CHWs] feel good standing there wearing a t-shirt, having a bag, an umbrella and gum boots. We appreciate the incentives we sometimes receive … that form of motivation emphasizes to us that we are still relevant and recognized … but also financial incentives to cover some expenses would enable us work better.* —CHW, Group 2

#### Stakeholder Engagement and Collaboration

Workshop participants stressed that continuous stakeholder engagement through regular meetings with all partners at central and local levels including CHWs was fundamental. In addition, stakeholders noted that involving communities in all CHW program processes (design, implementation, and evaluation) ensures community ownership. It was also emphasized that influential community persons such as local, religious, and traditional leaders are important in successful community entry and promoting health interventions in communities. Stakeholders also agreed that strategies that foster collaboration between implementing partners and the government both support and strengthen CHW programs. It was also stressed that public-private partnerships allow effective program alignment and integration with national health priorities, which is important for desirable service delivery. For instance, stakeholders highlighted that financial resources from districts and health facilities together with funding from development partners have substantial promise for program sustainability if collaboratively well planned for and used.

Stakeholders also agreed that strategies that foster collaboration between implementing partners and the government both support and strengthen CHW programs.

### Challenges Affecting CHW Programs

CHW programs face several challenges including poor coordination, fragmented data collection systems, high program expectations, inadequate support mechanisms, and high dropout rates.

#### Poor Coordination

Stakeholders emphasized that proper coordination of CHW programs provides great opportunities for planning and interactions that enable partners to share experiences. However, stakeholders reported poor coordination among implementing partners and the government, evidenced through inadequate communication, inconsistent and insufficient facilitation mechanisms, nonstreamlined workloads for CHWs, and implementation of parallel CHW programs. Besides having a few meetings with some partners, stakeholders felt that the MOH had not provided an adequate supportive environment for effective communication with all stakeholders, sometimes leading to contradictory information during implementation that affected progress and relevance of some programs. They reported that the lack of standard facilitation mechanisms for CHWs was common, with some development partners providing higher facilitation compared to government programs. Stakeholders said that this lack of uniformity in facilitating CHWs attracted most of them to prefer supporting nongovernmental than government programs. However, unfair operations among some development partners were noted. For example, CHWs reported that some partners provided insufficient transport refunds without considering the long distances CHWs traveled from hard-to-reach communities.

*They can invite you [CHW] for a meeting and give you UGX 2,000 [approximately 0.50 US$] a day as transport refund yet we [CHWs] come from far places where one may be actually using UGX 10,000 [approximately 3 US$] for transport which makes that refund very unfair.* —CHW, Group 1

#### Fragmented Data Collection Systems

There were concerns about the poor data quality, which was related to nonstreamlined reporting mechanisms among implementing partners including the government. Various partners used different reporting tools and indicators specific to their needs and funding requirements. Consequently, CHWs got burdened with many different reporting materials and processes, which reduced their efficiency and quality of reports. One NGO member described such challenges:

*Implementing partners … have their own reporting tools and indicators … yet other indicators that should be entered in the MOH reporting tool are left out. Therefore, that kind of parallel reporting is a problem.* —Member from an NGO, Group 3

#### High Program Expectations

Stakeholders felt that local leaders and communities had high expectations of CHW programs and various partners lacked honesty and transparency, which, in turn, raised expectations by communities and CHWs that many programs could not meet. For instance, beyond a program's objectives, some implementing partners' programs were perceived as a solution for all prevailing health challenges in the communities. Similarly, some communities had high expectations of CHWs, who they then perceived as being nonresponsive and underperformers regarding their responsibilities.

*CHWs are mandated to only treat children under the age of 5 years, but you can go to a family and they have a sick person beyond that age. For that reason, CHWs find it very difficult to treat such a person. Then community members say that these CHWs are not doing their job because of not treating such a patient.* —Health care provider, Group 3

#### Inadequate Support Mechanisms

Another challenge that CHWs faced was related to unsupportive systems as the overall support for CHWs from communities and the wider health system was reportedly limited. Support from key stakeholders, which is important for CHWs' performance, was inadequate and characterized by unequal treatment from some implementing partners, inappropriate reception at health facilities, and negative community attitudes. CHWs that were not involved in iCCM were less engaged in activities of most partners, which demotivated them as they felt less valuable and unrecognized in their communities. Many CHWs felt that the MOH and some health care providers did not value their role and contribution to health service delivery including when they attended health facilities. Similarly, the lack of proper community orientation about CHW roles was related to negative attitudes toward them.

Support from key stakeholders was inadequate and characterized by unequal treatment from some implementing partners, inappropriate reception at health facilities, and negative community attitudes.

*CHWs are trying to do a lot but it's not well recognized in our country. There is a challenge of CHW recognition and at times they find themselves not attended to when they go to the health facility because for example the nurse on duty claims, “I don't know you.” There is that attitude of giving them less attention, yet this is one of the things which would really motivate them to work hard. Another challenge is that there is unequal treatment where you will find CHWs fragmented and treated according to which partner is working with them.* —Member from an NGO, Group 2

#### High Dropout Rates

High dropout rates, particularly among young and male CHWs, was another challenge. Discussions revealed that many male and young CHWs dropped out to look for paying jobs soon after they had been recruited and trained. Some CHWs were reported to have too high expectations of the work despite it being a voluntary service. Stakeholders were concerned that these high dropout rates created gaps in the general delivery of community health services and that replacement of CHWs was costly to the program in terms of time and financial resources. Specifically, they noted that all new CHWs would need to undergo an initiation training, which was reportedly costly and therefore was not conducted as often as necessary.

*We had initially thought we would have younger CHWs. But when they come, you invest so much in training them and 3 months down the road they say, “I am going to look for a job.” Then you need to recruit other people and have to retrain again. So regarding youth participation in CHW programs, we have failed.* —District health team member, Group 1

### Enhancing Performance of CHWs

Discussions on ways to improve CHW performance emphasized strengthening recruitment, training, and retention strategies; improving motivation; streamlining coordination mechanisms; and developing and strengthening community health policies.

#### Strengthen Recruitment, Training, and Retention Strategies

To enhance CHW performance and retention, stakeholders recommended streamlining the recruitment criteria, which ensures active involvement of communities and proper communication of CHW roles to all stakeholders. Stakeholders said that CHW recruitment criteria should be improved and operationalized to avoid ambiguity. Standardizing capacity building by using uniform training materials and appropriate technology was also recommended to help CHW performance and retention.

Stakeholders stressed that the need to ensure more males and young people are involved in CHW programs. They noted that male CHWs were able to support community mobilization and contribute significantly to the health of men, while young people would be well-positioned to get involved in youth-friendly services including engaging their peers. Therefore, they recommended considering relevant and responsive strategies for innovative recruitment of CHWs catered to increasing gender and age diversity.

*Most CHWs are women, as well as being 40 years and above therefore there is need for more diversity. We have struggled to have more young people engaged hence lack young CHWs who can interest young people to participate in health initiatives. We need to devise means to actively increase youth and male engagement for the betterment of health of the communities.* —MOH official, Group 1

#### Improve Motivation of CHWs

Stakeholders noted that the availability of incentives (financial and nonfinancial), enhanced transportation, manageable workload, and adequate support and engagement were important elements for CHW motivation. They also noted the importance of providing nonfinancial incentives to CHWs through recognition at important community events and showing CHWs appreciation with certificates after training sessions. In addition, stakeholders had strong opinions that providing regular financial incentives for CHWs would be essential in improving their performance. Stakeholders recommended providing CHW coordinators with motorcycles to enhance transportation, which would improve supervision including timely delivery of supplies and collection of reports. Similarly, ensuring adequate numbers and distribution of CHWs in all villages would reduce their workload as well as distances traveled to communities. Additionally, the provision of meals and reimbursement of transport costs whenever CHWs were invited for trainings and other activities was seen as crucial to avoid the financial difficulty that they often find themselves in.

*When a CHW is moving from one village to another, they are taking off time to serve. Many times, they have to use their own resources if they need a meal during their work. So, what we should advocate for is that CHWs should be financially facilitated while doing community health work. Therefore, whatever cost they incur while doing their work should be reimbursed. We need to strike that balance regarding how much voluntary work they can do without much support.* —Member from an NGO, Group 3

Discussions also revealed that guaranteeing adequate support for and involvement of CHWs to improve program inclusiveness by implementing partners is vital for their motivation. Many stakeholders said that any unfair engagement regarding incentives or training activities can potentially demotivate CHWs. For example, they recommended that all CHWs (regardless of involvement in iCCM) should be equally involved and supported to play their role in improving community health.

Many stakeholders said that any unfair engagement regarding incentives or training activities can potentially demotivate CHWs.

#### Streamline Coordination Mechanisms

**Proper planning, transparency, and accountability**. Proper coordination of all stakeholders at national and district levels was recommended to ensure appropriate planning, transparency, and accountability of CHW programs. Regular meetings of all implementing partners were believed to be an opportunity to present and share progress that would avoid miscommunication, varying compensation policies, unnecessary expectations among stakeholders, and duplication of efforts. At the community level, stakeholders noted that strong coordination also ensures effective mobilization of CHWs and communities including political leaders.

*There is need to streamline district coordination and planning and hold quarterly meetings to review what has been done which will ensure transparency of all partners.* —Member from an NGO, Group 2

**Support supervision of CHWs.** Stakeholders noted that strengthened supervision by the MOH and districts by providing appropriate leadership, including overseeing all activities conducted by implementing partners, would enable CHWs to improve their performance. Stakeholders also agreed that efforts such as consistent follow-up of activities and regular refresher trainings to ensure adherence to standardized protocols can reinforce overall supervision and should be prioritized.

Many stakeholders strongly articulated a need to prioritize harmonizing data collection and management by standardizing tools and digitalizing reporting systems to ensure real-time data collection and reporting. Stakeholders agreed that uniform reporting tools were an opportunity for improving data quality, reducing CHW workload, and supporting systematic program monitoring by districts and the MOH. The significance of aligning various technologies such as mobile digital applications, used by different partners to avoid discrepancies and delays in reporting, was also emphasized as being key for greater impact of CHWs on the wider health system.

*There is fragmentation…. partners use different technologies and applications… I think more integration of technologies is required, particularly by the MOH. Maybe the ministry can recommend a standard technology and application so that monitoring of CHW work by all implementing partners is easier and consistent.* —Member from an NGO, Group 3

#### Develop and Strengthen Community Health Policies

Recognizing that CHWs cannot do much when they are not fully supported by the national health system, stakeholders called for active and fruitful engagement of policy makers in CHW programs. Stakeholders highlighted the need for developing new policies or strengthening existing ones to streamline broader concerns of training, supervision, and motivation of CHWs. The issue of inadequate equipment and supplies was emphasized, and stakeholders called for policies aimed at ensuring consistent availability of supplies such as drugs necessary to improve CHW performance. In addition, stakeholders called for better implementation of such policies as opposed to developing good ones that remain nonoperational.

Recognizing that CHWs cannot do much when they are not fully supported by the national health system, stakeholders called for active and fruitful engagement of policy makers in CHW programs.

### Ensuring Sustainability of CHW Programs

Deliberations from the workshop regarding the sustainability of CHW programs at national, district, and community levels stressed the need for institutionalization of CHW programs, sustainable funding, economic empowerment of CHWs, local ownership of CHW programs, and strengthened CHW research agendas.

#### Institutionalization of CHW Programs

Institutionalization of CHW programs was important for strengthened partnership and collaboration among all stakeholders that should be focused on effective supervision, performance monitoring, and good management (including transparency and accountability). Stressing the importance of clear government leadership for all CHW programs (even if they use different implementation models), stakeholders emphasized that different partners should work within government structures for enhanced sustainability.

Besides improved national coordination, it was noted that institutionalization is significant for continuous steering of program implementation, prioritization, and funding for key interventions, as well as creation of a strong foundation for sustainability. The need for a national CHW forum was suggested to allow for the regular engagement of stakeholders (including community members and CHWs) to share experiences, challenges, and progress of various interventions. This forum would also be used to discuss strategies to continuously advance the role of CHW programs in addressing the current and future communicable and noncommunicable disease burden and other health concerns in the country.

#### Sustainable Funding

The importance of ensuring sustainable funding for CHW programs by the MOH and implementing partners was a key area of debate during the workshop. Most stakeholders emphasized that by increasing budget allocations at national and district levels for CHW programs, the government can strengthen its ownership and commitment and accrue significant community health benefits.

*Government providing more resources for the CHW program is something that we should advocate for because CHWs greatly support community health, know the communities very well, understand the people, and can better identify many community problems because they live within these communities.* —Member from an NGO, Group 1

Pooled funding by implementing partners, which was believed to be more sustainable than disaggregated support, was strongly recommended. The stakeholders agreed that it was important for resources to be pooled together that can then be appropriately managed to equitably support CHW programs. It was also noted that collaborative resource mobilization benefits communities and CHWs, builds sustainable programs, and increases capacity to advocate for further support including partnerships.

#### Economic Empowerment of CHWs

Stakeholders suggested 2 major mechanisms that can help CHWs feel more empowered and could be used to develop a strategy to sustain their involvement given the lack of remuneration they suffer: (1) establish CHW-focused savings and credit cooperative organizations to enhance their access to financial credit for income-generating activities; and (2) train CHWs in entrepreneurship to develop hands-on skills (e.g., tailoring and improved farming practices) to enhance their productivity to sustainably meet economic demands.

*CHWs too need money to meet their personal and household needs. And if they have easy access to savings and credits groups, may be even own their own group where they are members and would easily get affordable loans, could help their development hence supporting them to do community work. Of course, teaching them other income-generating activities, for example, farming would also be beneficial for sustaining themselves financially.* —Community member/leader, Group 3

#### Local Ownership of CHW Programs

Program ownership by communities, CHWs, and district health teams is considered central for sustainability. The stakeholders discussed the following 2 major mechanisms for ensuring that communities owned and sustained CHW programs.

**Transparent implementation and community sensitization.** Stakeholders agreed that transparent and accountable implementation during all processes, including CHW recruitment and deployment, was key in building community trust. Building strong ownership of programs and undertaking community sensitization (including among local leaders) on the role of the CHW and the public's expectations would help to create more positive attitudes and recognition of their work. Stakeholders also agreed that the local government and health care providers should identify and use opportunities during community gatherings to sensitize people about CHW programs. Other strategies that have the potential to increase awareness and ownership of CHW programs included a public-private partnership with relevant agencies, such as communication companies that could support community sensitization.

**Increased CHW involvement and streamlined program benefits.** Discussions highlighted the need for increased CHW involvement in their programs from design to evaluation. Such involvement would warrant CHWs' sustained commitment by reconciling their expectations, demands, roles, and work mechanisms with all implementing partners. To ensure that CHWs can remain relevant, are retained, and sustained in the long term, stakeholders stressed the need for streamlined benefits such as a career path including alignment with government-wide initiatives. For instance, stakeholders recommended a priority consideration for upgrading qualifying CHWs into new programs such as the proposed national community health extension workers (CHEWs) program to motivate them and highlight their potential career growth.

*Can we have CHWs move from just being volunteers … I have not seen it but I would love to see some of them growing in their career, skills and becoming maybe something higher than when they joined the program? That is career growth, something that would be of interest and benefit them as well.* —Member from an NGO, Group 3

The introduction of a community health insurance scheme was also discussed as part of streamlining wider CHW program benefits. CHWs themselves have health needs that they must meet, and stakeholders were concerned about the potential challenges in maintaining their own health while voluntarily promoting the health of others in communities. Developing community health insurance initiatives to reduce health-related economic consequences among communities, with additional benefits for CHWs as a form of motivation, was noted to be essential for program sustainability. Stakeholders strongly felt that community health insurance, with benefits for CHWs such as cost-subsidized services, would empower and motivate them to work with less worry about their own health demands and related expenses.

#### Strengthened CHW Research Agenda

The stakeholders called for strengthening research focusing on CHWs to contribute to evidence-based strategies for sustained community health programs. They stressed that CHW programs should be driven by data in all aspects of design, implementation, and evaluation—which should all incorporate research. However, stakeholders noted that many questions remain unanswered, including the amount of financial resources required to effectively implement CHW programs in Uganda which could be answered through research. Workshop proceedings also revealed a requirement for holistic support for research institutions to ensure that there is the required expertise, funding, and favorable environments, as well as infrastructure to conduct responsive studies for CHW programs in the country.

*If you were to ask what budget is needed to implement a CHW program in one subcounty, no one would probably ably attempt that question … so, I think you have brought in the element of research and the need to give out an investment case [*for CHW programming*], and this is where universities need to help with research, because we NGOs, that is something important for us, to know the cost of engaging CHWs.* —Member from an NGO, Group 1

All emerging workshop themes and subthemes are summarized in Table 2.

**TABLE 2.** Key Themes and Subthemes From a 1-Day Workshop on the Uganda CHW Program

Lessons Learned in Implementing CHW Programs	Challenges Affecting CHW Programs	Strategies to Improve CHW Performance	Ensuring Sustainability of CHW Programs
Capacity building and use of technology	Poor coordination	Strengthen recruitment, training, and retention strategies of CHWs	Institutionalization of CHW programs
Supervision and motivation	Fragmented data systems	Improve motivation of CHWs	Sustainable funding
Stakeholder engagement and collaboration	High program expectations	Streamline coordination mechanisms	Economic empowerment of CHWs
	Inadequate support mechanisms	Develop and strengthen community health policies	Local ownership of CHW programs
	High dropout rates		Strengthened CHW research agenda

Abbreviation: CHW, community health worker.

## DISCUSSION

This study explored stakeholders' lessons and experiences in implementing and supporting CHW programs in Uganda. Whereas concerns of training, supervision, and motivation have long been known to affect CHWs,^12^^,^^16^^,^^17^ our findings emphasize the importance of technology as well as stakeholders closely working together in improving CHW programming. CHWs in Uganda are lay community members whose capacity ought to be built to fully understand their roles and responsibilities and provide them with the information and skills they require. The capacity-building avenues include initiation and refresher training and supportive supervision whose relevance has previously been emphasized.^7^^,^^11^^,^^17^^,^^20^^,^^21^ Previous studies have also underscored the importance of supportive supervision for CHWs by health care providers, including accompaniment and shadowing, which gives them important feedback and confidence to perform their roles, improves their acceptability, and builds community trust.^22^^–^^24^ Although not fully explored in Uganda, capacity building of CHWs using technology has been recognized for facilitating other tasks, such as data collection and reporting, and improving outcomes, such as CHW motivation and empowerment. A systematic review that evaluated program outcomes when using mobile tools noted that mobile technologies support CHWs to receive alerts and reminders, facilitate health education sessions, and conduct person-to-person communication. ^25^ This review noted that mobile tools helped CHWs to improve the quality of care provided, the efficiency of services, and the capacity for program monitoring, thus presenting a key opportunity for CHW programs. Therefore, it is prudent for MOH and other stakeholders in Uganda supporting CHWs to fully embrace technology as a tool that can improve CHW performance and outcomes.

The role of motivation from both financial and nonfinancial incentives in CHW programs cannot be overemphasized. Previous studies conducted in Uganda suggest that CHWs valued nonfinancial incentives more than financial ones.^26^^,^^27^ However, financial incentives continue to be emphasized by CHWs and other stakeholders such as implementing partners supporting CHWs programs.^20^^,^^21^^,^^24^ The World Health Organization, through their guidelines on health policy and system support for optimizing CHW programs, recommends that practicing CHWs should be recognized as part of the workforce and provided with a financial package in line with assigned duties, training, roles, and working hours.^28^

Our study also highlighted the relevance of CHW stakeholder engagement and collaboration at different levels, including with the community. Involvement of all actors leverages available resources, influences the acceptability of programs, and enhances community buy-in and ownership.^9^^,^^24^^,^^29^ Stakeholder involvement also streamlines planning and effective service delivery^9^^,^^24^^,^^29^ in line with national policies and should be strengthened and incorporated within community health programming. Opportunities for stakeholders interacting often as well as jointly participating in planning, implementation, and evaluation of CHW programs would have benefits in improving community health.

Our study noted several challenges that affect CHW programs in Uganda including poor coordination, fragmented data collection systems, high program expectations, inadequate support mechanisms, and high dropout rates. Similarly, a 2014 Uganda survey highlighted differences in implementation of the strategy and found several gaps in supervision, motivation, and coordination of programs.^30^ Many of these challenges have also been reported by previous studies conducted in the country.^11^^,^^21^^,^^31^^,^^32^

From our study, the need for younger CHWs and males to support the CHW program was highlighted as previously observed.^33^^,^^34^ Even when identified and trained, attrition among these groups is usually high, which can be attributed to the unattractive CHW voluntary role, as males are the main income earners and young people usually search for better opportunities.^35^^,^^36^ However, the World Health Organization CHW guidelines reported no specific evidence of the relevance of age and gender as selection criteria for CHWs and recommended that recruitment and selection procedures should prioritize other criteria such as relevant life experience, acceptability, caring attitude, commitment, and other individual attributes.^28^ Nevertheless, the guidelines recommend maximizing women's participation and promoting their empowerment as well as considering the sociocultural context of operation.^28^ Our earlier study established that although there were more female CHWs, male CHWs could perform some roles while supporting their communities.^33^ These roles include quick response to emergencies, community mobilization for public health interventions, and activities involving manual labor such as protecting water sources. The challenges affecting CHW programs related to gender and age ought to be readily addressed to improve the diversity and effectiveness of Uganda's CHW programs. Certainly, having a good mix of age and gender for CHWs is likely to improve service delivery in communities.

The challenges affecting CHW programs related to gender and age ought to be readily addressed to improve the diversity and effectiveness of Uganda's CHW programs.

To enhance the performance of CHW programs, stakeholders suggested the need for strengthened recruitment, training and retention strategies, improved motivation avenues, streamlining of coordination mechanisms, and development and strengthening of community health policies. These recommendations are in line with those provided by previous studies in Uganda.^11^^,^^17^^,^^21^^,^^22^^,^^32^ In addition, the Uganda MOH-commissioned review of the CHW strategy recommended the need to strengthen the program with clear recruitment, funding, coordination, motivation, and supervision mechanisms.^30^ Thereafter, the MOH suggested plans to introduce CHEWs as a paid cadre of CHWs and prescribed their functionality mechanisms. The CHEWs would be based at the parish level and support the work of the existing CHWs (VHTs) including supervision, data collection and reporting, and diagnosis and management of simple illnesses.^37^ However, it has been argued that the CHEWs approach may not be adequately funded, will face practical and logistical implementation challenges, and could create tension with the current CHWs and community.^38^ Therefore, there is a need to rethink an effective and contextual community health strategy for Uganda to deal with the current challenges and guide the institutionalization of the program to enable CHWs to achieve their full potential and spur public health benefits.

Beyond performance, stakeholders highlighted measures to enhance the sustainability of CHW programs. These measures included fully institutionalizing CHW programs, which should involve engaging with and empowering communities, implementing national programs at scale, ensuring sufficient and sustainable financing for community health systems, and integrating community data into the health information system.^39^ In addition, effective program design and management, its fit with specific communities served, and integration within the broader political, economic, and health system environment are key factors for scaling up and sustaining CHW programs in LMICs.^40^ Institutionalization should improve national coordination of CHW programs led and directed by the government, create mechanisms for sustainable funding of programs, and ensure their local ownership as suggested by our study rather than dependency on external donor-led programs. Since the CHW program in Uganda currently depends on voluntary labor, alternative income-generation streams should be established to support CHWs to meet their financial needs. In reality, CHW livelihoods are similar to that of the communities they serve, and they too are vulnerable to shocks such as food insecurity aligned with poor coping mechanisms.^41^ Thus, measures such as the establishment of savings and credit cooperative organizations and equipping CHWs with entrepreneurship skills as suggested in our study are important to empower them to improve their livelihoods. To further improve the sustainability of CHW programs, the research agenda on CHWs as established in our study needs to be strengthened. Research needs to be incorporated into all aspects of CHW programming to provide opportunities for learning and system improvements. Research on CHW programming could also provide preliminary evidence before implementing large-scale programs and incorporating continuous process improvements. The role of the MOH, implementing partners, and academic institutions remain paramount in building capacity, finding sustainable funding streams, and creating a favorable environment to advance the CHW research agenda in Uganda.

### Strengths

A key contribution of this article to the literature is the use of workshop methodology in health systems research. Although workshops are not a traditional method for engaging participants and collecting data, their use has increased in recent years.^42^^–^^44^ As our study demonstrated, some of the benefits of using workshops include bringing together a diverse range of individuals to generate data for research, obtaining a wealth of data on a topic of interest in a short period, and learning from others' experiences during the process, which is not possible using other data collection methods. Given that the stakeholders who participated in the workshop were working in various parts of Uganda, the results from the study provide a general representation across the country. However, key concerns regarding the use of workshops include power dynamics ^45^ and positionality.^46^ Unlike focus group discussions that may have participants of the same social class or cadre, a workshop may have a diverse range of individuals involved. As an example, our workshop participants included policy makers, implementers including health care providers, researchers, and CHWs. For this reason, power dynamics need to be considered during the workshop planning as it could dictate the extent of participant engagement.^47^ Indeed, certain participants (such as community members) may not openly express their views in the presence of high-ranking officials some of whom may be their supervisors or superiors. We addressed this concern by emphasizing that the workshop was solely for research purposes and encouraged participants to feel free to share their views and experiences. In addition, we advised the group moderators to ensure all participants contributed to the discussions hence avoiding domination by a few individuals. Regarding positionality, the role that the researchers play in the workshop process can influence the outcome of the activity including the level of participant involvement. In our study, we ensured the researchers did not moderate any of the group discussions and their contribution was minimal to minimize the researchers' influence on the workshop proceedings. However, other researchers have shared their personal stories as part of workshops to situate themselves in a more equal position with participants.^46^ Nevertheless, such a level of engagement of researchers in discussions may best suit a workshop that has 1 cadre of participants. With these benefits and concerns in mind, the workshop methodology can be explored by other health systems researchers as a means of engagement and discussion with various individuals collectively on a subject of interest.

### Limitations

Although we made the workshop as inclusive as possible, stakeholders from some organizations did not attend. Future related studies may explore using a framework that combines performance and sustainability as a basis to inform the analysis and subsequent discussion.

## CONCLUSION

To improve the performance and sustainability of CHWs programs, stakeholders such as policy makers including MOH officials, district health authorities, and implementing partners, need to consider CHW needs, existing structures, and policies, as well as local ownership and support. The workshop methodology can be used in the future in health systems research to inform policy, practice, and programming, particularly in LMICs.

## Supplementary Material

21-00260-Musoke-Supplement-clean.pdf

## Notes

**Peer Reviewed**
