# Supplementary material for: Enhancing Performance and Sustainability of Community Health Worker Programs in Uganda: Lessons and Experiences From Stakeholders
Source: Glob Health Sci Pract. 2021 Dec 31;9(4):855–68. doi: 10.9745/GHSP-D-21-00260 (PMC8691894; doi:10.9745/GHSP-D-21-00260)
Supplement: 21-00260-Musoke-Supplement-clean.pdf [file 21-00260-Musoke-Supplement-clean.pdf]

Supplement to: Musoke D, Atusingwize E, Ndejjo R, et al. Enhancing performance and sustainability of community health worker programs in Uganda: lessons and experiences from stakeholders. *Glob Health Sci Pract*. 2021;9(4). https://doi.org/10.9745/GHSP-D-21-00260

Stakeholder Workshop on the Community Health Worker Program in Uganda

The 1-day workshop conducted comprised 3 sessions. The first session involved presentation of key lessons learned about CHW programs from 4 selected implementing partners. The 4 partners that shared experiences of supporting CHWs programs in Uganda were: the NTU-MakSPH partnership, BRAC, World Vision, and Living Goods that all operated in different regions and contexts across the country. For example, the NTU-MakSPH partnership was supporting CHWs in Wakiso district in the central region of the country with a focus on training, supervision, and motivation, while Living Goods was empowering CHWs to deliver lifesaving products to communities at relatively low cost in 20 districts in Eastern and Northern Uganda. BRAC operated in 87 districts in the country working with CHWs to improve economic and health status of communities especially children under 5 years of age, adolescent girls, and pregnant women. World Vision's program focused on improving maternal and child health, nutrition empowerment of communities, and improved access to HIV, water, and sanitation services in Eastern and Northern Uganda.

The second session of the workshop involved group discussions that focused on critical aspects of CHW programs. The discussions were carried out in 3 groups that were constituted to ensure diversity in terms of stakeholder affiliations. Each group had representation from policy makers (MOH), local government (district health office or health practitioner), implementers / NGOs, academia (researcher or student), and the community (CHWs). The average number of participants per group was 15. The discussions were guided by 3 themes which were developed based on key elements of CHW programs in Uganda using the over 10-years' experience of the researchers in supporting CHWs in the country and other parts of the world, as well as existing literature. Below are the themes which were explored by all groups:

1. Experiences of working with communities for health improvement including what has worked, challenges faced, and how they have been addressed.

2. Enhancing support to CHWs to improve their performance in health service delivery considering their recruitment, training, retention, supervision, motivation, reporting, transportation, equipment and supplies, and use of technology.

3. Increasing sustainability of CHW programs in Uganda considering funding, enabling environment, local government engagement and support, collaboration, monitoring and evaluation, research, innovations and learning fora.

The third session of the workshop was held as a plenary, where all groups provided feedback from their respective discussions. Each group identified a moderator who guided the discussion, and an assigned note taker supported the documenting of all the workshop proceedings including the group and plenary sessions. Two of the moderators were academics while one was from an

NGO. During the plenary session, key emerging issues were discussed, with respective groups responding to emerging questions.
